# The Relationship between Obesity, Prostate Tumor Infiltrating Lymphocytes and Macrophages, and Biochemical Failure

**DOI:** 10.1371/journal.pone.0159109

**Published:** 2016-08-03

**Authors:** Charnita Zeigler-Johnson, Knashawn H. Morales, Priti Lal, Michael Feldman

**Affiliations:** 1 Division of Population Science, Department of Medical Oncology, Thomas Jefferson University, Philadelphia, Pennsylvania, United States of America; 2 Center for Clinical Epidemiology and Biostatistics, University of Pennsylvania, Philadelphia, Pennsylvania, United States of America; 3 Department of Pathology, University of Pennsylvania, Philadelphia, Pennsylvania, United States of America; "INSERM", FRANCE

## Abstract

**Background:**

Obesity reflects a chronic inflammatory environment that may contribute to prostate cancer progression and poor treatment outcomes. However, it is not clear which mechanisms drive this association within the tumor microenvironment. The aim of this pilot study was to examine prostatic inflammation via tumor infiltrating lymphocytes and macrophages characterized by obesity and cancer severity.

**Methods:**

We studied paraffin-embedded prostatectomy tissue from 99 participants (63 non-obese and 36 obese) from the Study of Clinical Outcomes, Risk and Ethnicity (University of Pennsylvania). Pathologists analyzed the tissue for type and count of lymphocytes and macrophages, including CD3, CD8, FOXP3, and CD68. Pathology data were linked to clinical and demographic variables. Statistical analyses included frequency tables, Kruskal-Wallis tests, Spearman correlations, and multivariable models.

**Results:**

We observed positive univariate associations between the number of CD68 cells and tumor grade (p = 0.019). In multivariable analysis, CD8 counts were associated with time to biochemical failure (HR = 1.09, 95% CI = 1.004–1.192, p-value = 0.041.) There were no differences in lymphocytes or macrophages by obesity status or BMI.

**Conclusions:**

The number of lymphocytes and macrophages in the tumor microenvironment did not differ by obesity status. However, these inflammation markers were associated with poor prostate cancer outcomes. Further examination of underlying mechanisms that influence obesity-related effects on prostate cancer outcomes is warranted. Such research will guide immunotherapy protocols and weight management as they apply to diverse patient populations and phenotypes.

## Introduction

Obesity is a potentially modifiable risk factor for disease progression and poor outcomes for numerous diseases, including prostate cancer (PCa). Although not all studies support a relationship [1–5], it is generally believed that obesity increases the risk for advanced PCa stage and grade at diagnosis, younger age at diagnosis, biochemical failure (disease recurrence) after treatment, and PCa-specific mortality [5–9]. However, the mechanisms by which obesity affects poor PCa outcomes are not understood.

One possible link in the relationship between obesity and PCa progression is inflammation. Obesity produces a state of systemic chronic low-grade inflammation that can contribute to a number of chronic diseases, including advanced PCa [9–13]. In recent years, researchers interested in the underlying biology of carcinogenesis and metastasis have investigated the tumor microenvironment, which typically displays an inflammatory nature [14]. They have observed immune infiltrates in various solid tumors of different patient populations [15]. Chronic pathologies are mainly comprised of tumor infiltrating lymphocytes (TILS) and tumor associated macrophages (TAMS). [9] The most abundant immunologic cell types within the microenvironment appear to be TAM CD68 and TIL CD3 with subtypes CD8 and FOXP3. CD3 is expressed on all TILs, while some TILs also express CD8 (“killer” cells) or FOXP3 (linked to CD4 “helper” cells). CD68 is expressed on macrophages. Under chronic inflammation, TILS and TAMS in the tumor microenvironment secrete various factors that may increase cell proliferation and inhibit cell death, potentially advancing cancer. [13,16] The prognostic value of these cells is still under investigation; however the presence of these immune cells in tumor samples may indicate aggressive tumors that are likely to metastasize. In obese patients, the immune system may be further compromised and, as a result, an increased presence of immune cells may be found in tumor microenvironments [17,18].

We have previously reported that obesity is associated with poor PCa outcomes [6]. However, variation in TILS and TAMS by obesity status has not been described. The goal of this study was to examine PCa specimens to characterize differences in TILS and TAM within the tumor microenvironment by obesity status and cancer severity. We hypothesized that these tumor infiltrates would be more prevalent in obese patients compared to non-obese and in patients with more aggressive cancers compared to less aggressive cases.

## Materials and Methods

The current study was approved by the Institutional Review Board at the University of Pennsylvania. Written informed consent was obtained from participates in the Study of Clinical Outcomes, Risk, and Ethnicity (SCORE).

### Study Sample

Prostate tissue specimens were selected from patients identified from SCORE between 1995 and 2015 and treated with radical prostatectomy in the University of Pennsylvania Health System [19,20].

PCa case status was confirmed by medical records review using a standardized abstraction form. All invasive PCa cases with incident (within 24 months of diagnosis) pathologically diagnosed tumor were eligible for inclusion in this study. Men were excluded if they reported having exposure to finasteride or dutasteride at any time prior to their diagnosis. Men were also excluded if they had ever been diagnosed with cancer at any site other than their recently diagnosed PCa.

Tissue samples from 150 patients (75 normal weight vs. 75 obese patients) were selected for inclusion in the study, based on SCORE race/ethnicity distribution and obesity status. Patient weight and height were obtained from medical record abstraction and used to compute body mass index (BMI). Normal weight was defined as BMI at diagnosis < 25kg/m^2^. Obesity was defined as BMI at diagnosis ≥ 30kg/m^2^.

### Pathology

**Fig 1:**
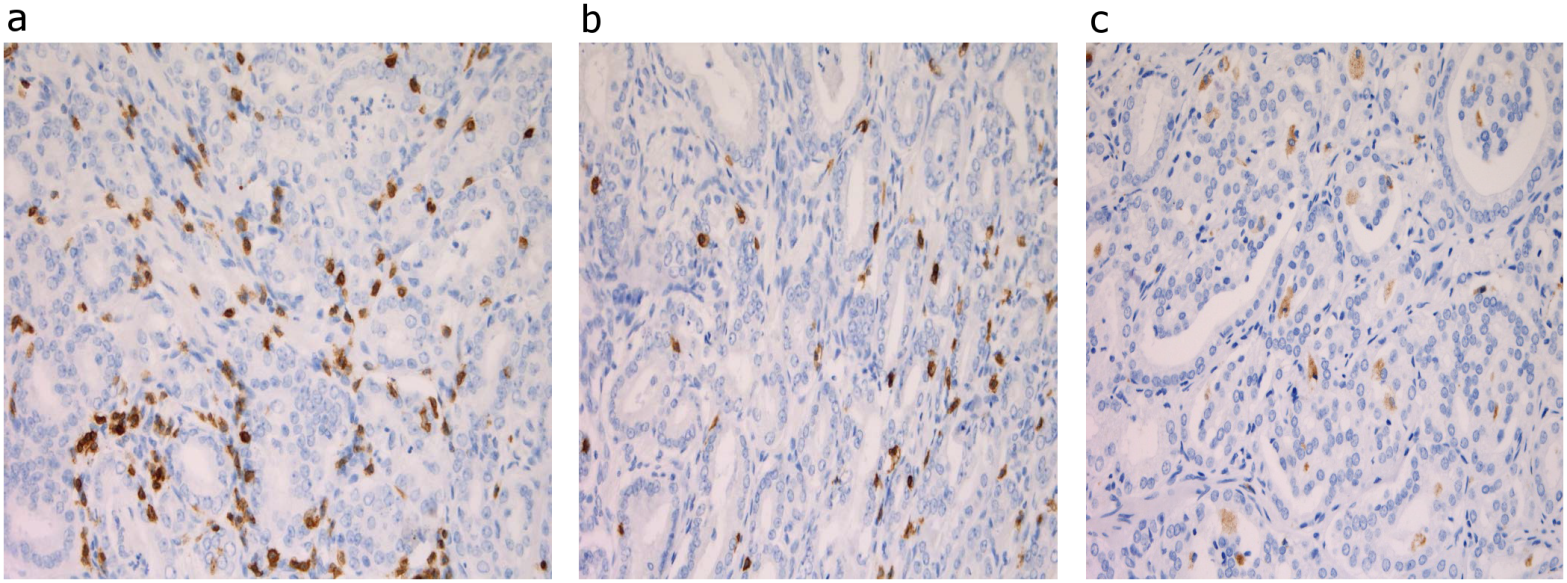
Representative pictures of stainings for tumor infiltrating immune cells: a) CD3 b) CD8 c) CD68.

To characterize prominent prostate tumor infiltrates, we focused on abundant immunologic cell sub-types. We cut, stained and imaged archived formalin-fixed paraffin-embedded (FFPE) tumor tissue samples from the SCORE cases. Then we analyzed their cell type and count in the Department of Pathology and Laboratory Medicine at the University of Pennsylvania. Prostate sections from resected glands were stained for cytokeratin from tumor cells and T cells (CD3, CD8, FoxP3) and macrophages (CD68.) (Fig 1) The antibody clones that we used were CD3: clone LN 10 (Leica, catalog #NCL-L-3); CD8: clone C8/144B (DAKO, catalog #M7103); FOXP3: clone 206D (Biolegend, catalog #320102); and CD68: clone KP1 (DAKO, catalog #IR60961). The entire tumor nodule was scanned at low power to survey overall prevalence of infiltrating immune cells. We then selected 4 representative fields from each tumor sample. The area of the tumor that was selected depended on where the majority of cells were found in each tumor sample. Tissues were scored for infiltrating lymphocytes and macrophages in the dominant nodule (largest tumor nodule identified in the prostate) [21] by manually counting the number of TILs or TAMs in 4 fields at 20X for T cells and macrophages and averaging the scores.

### PCa Outcomes

Data related to clinical characteristics, tumor pathology, and PCa outcomes were collected via medical record abstraction. The primary study outcomes were advanced tumor stage (T3 or T4) and tumor grade (≥ 7) at diagnosis, and biochemical failure. Biochemical failure was evaluated as PCa recurrence after prostatectomy, defined as a single PSA ≥ 0.2 ng/mL or two consecutive values of PSA = 0.2 ng/ml. Biochemical failure data were obtained prospectively after prostatectomy. Follow-up information about biochemical failure was documented by rising prostate specific antigen (PSA) levels and obtained on an approximately 6-month basis by review of medical records. Selected cases were followed for a median 32 months (range = 3–147 months) to determine biochemical failure.

### Analytical Methods

To compare patient characteristics by obesity status, we made contingency tables for categorical variables and computed medians for continuous measures. A formal test of differences by obesity status was conducted using Kruskal-Wallis and Fisher’s exact tests. Correlation between the pathology measures was examined using Spearman correlation coefficients. Obesity status and PCa stage and grade at diagnosis were related to continuously, non-normally distributed CD68, CD3 and CD8 counts also using Kruskal-Wallis tests. Associations with the presence or absence of FOXP3 were examined using Fisher’s exact test.

The association between time to biochemical failure and tumor infiltrates was examined using a Cox proportional hazard model. The model included CD3, CD8, CD68, FOXP3, age, race, and tumor grade. A test of the proportional hazards assumption was performed based on the Schoenfeld residuals. [22]

## Results

Of the 150 selected samples, tissue from 63 normal weight (84%) and 36 obese patients (48%) were successfully evaluated for tumor cell infiltrates (total n = 99). The remaining samples could not be evaluated because the tissue specimens were insufficient after being used for other studies.

The tissue samples that were evaluated came from patients 42 to 73 years of age (median = 60 years). While 18.6% of these samples came from African American patients, the rest came from Caucasian patients. Obese and non-obese patients were similar on all demographic and clinical factors, except obese men were more likely to have been diagnosed with diabetes (25% vs. 3%, p = 0.001, Table 1.)

**Table 1:** SCORE demographics of obese and non-obese patients (N = 99).

Patient Characteristics	Non-obese (N = 63)	Obese (N = 36)	Kruskal-Wallis or Fisher’s exact p-value
Median Age, years (IQR)	61 (56–64)	58 (54–62)	0.065
African American, (%)	9 (14.29)	10 (27.78)	0.101
Married, (%)	51 (83.6)	30 (83.3)	0.972
Education, (%)			
<College	24 (39.3)	17 (48.6)	0.652
College	19 (31.2)	10 (28.6)	
Post-Graduate Degree	18 (29.5)	8 (22.9)	
Smoking Status, (%)			
Never	33 (56.9)	15 (42.9)	0.423
Former	20 (34.5)	16 (45.7)	
Current	5 (8.6)	4 (11.4)	
Median Body Mass Index, kg/m^2^	23.7 (23–24)	31.9 (31–34)	**<0.001**
Comorbidity Present, (%)			
	27 (42.9)	19 (52.8)	0.341
Diabetes	2 (3.2)	9 (25.0)	**0.001**
Tumor Stage, (%)			
T3/T4	23 (37.1)	18 (50.0)	0.212
Tumor Grade, (%)			
≥ 7	23 (46.9)	19 (65.5)	0.112
Median follow up time, months (IQR)	38 (19–63)	23 (12–59)	0.080
Biochemical Failure, (%)	10 (16.4)	11 (31.4)	0.086

Overall, the median CD3 cell count was 32 (range, 5–90); CD8 cell count was 16 (range, 4–47); and FOXP3 cell count was 1 (range, 0–15). FOXP3 cells were present in 75% of tumor samples and absent in the remaining 25%. Median CD68 cell count was 10 (range, 0–42). The CD3 cell count positively correlated with that of CD8 (r = 0.470, p < 0.001) and FOXP3 (r = 0.237, p = 0.037). No other significant correlations were observed.

There were no significant differences in bivariable analyses in immune cell counts by obesity status (Table 2) or diabetes status. However, we observed greater CD68 cell counts were associated with advanced tumor grade, regardless of obesity status (p = 0.019, Table 3).

**Table 2:** Tumor Infiltrating T Lymphocyte and Macrophage Count Associations with Obesity Status reported as median counts (interquartile range) unless otherwise specified.

TIL	All N = 97	Non-Obese N = 62	Obese N = 35	Kruskal-Wallis or Fisher’s exact p-value
T Cells				
CD3	32 (5–90)	33 (5–90)	30 (8–57)	0.182
CD8	16 (4–47)	16 (4–47)	18 (5–38)	0.289
FOXP3*	1 (0–14.6)	1 (0–14.6)	0.8 (0–13.5)	0.922
Presence of FOXP3, %*	74.7	75.0	74.1	0.928
Macrophages				
CD68	10 (0–42)	9 (2–27)	10 (0–42)	0.290

*Total N = 79

**Table 3:** Univariate Associations between Tumor Infiltrates and Prostate Cancer Outcomes: Median counts (interquartile range) unless otherwise specified.

TIL	Stage 1/2 N = 56	Stage 3/4 N = 41	Kruskal-Wallis or Fisher’s exact p-value	Grade <7 N = 36	Grade ≥7 N = 41	Kruskal-Wallis or Fisher’s exact p-value
T Cells						
CD3	34 (24–40)	30 (20–41)	0.336	35 (24–44)	30 (20–40)	0.335
CD8	16 (12–22)	17 (13–24)	0.251	15 (13–21)	19 (12–24)	0.207
FOXP3*	0.9 (0–3)	0.8 (0–5)	0.976	0.4 (0–2)	0.8 (0–3)	0.663
FOXP3 Presence, %*	79	71	0.434	67	68	0.935
Macrophages						
CD68	10 (5–16)	10 (7–17)	0.454	9 (6–13)	13 (8–17)	**0.019**

*Total N = 78 for stage; 61 for grade

Cox regression models showed no significant associations in the relationship of tumor infiltrates with biochemical failure including age, race, tumor grade, and individual infiltrate counts. However, models that included age, race, grade, CD3, CD8, FOXP3, and CD68 showed that only CD8 was associated with time to biochemical failure (Table 4). Higher CD8 counts were associated with an increased risk of biochemical failure.

**Table 4:** Multivariable Analysis for Tumor Infiltrates and Risk for Biochemical Failure in SCORE.

Outcome	Risk Factors	Hazard Ratio	95%CI	p-value
Time to Biochemical Failure	CD3	0.95	0.897–1.011	0.110
	CD8	1.09	**1.004–1.192**	**0.041**
	FOXP3	0.83	0.592–1.170	0.291
	CD68	1.03	0.966–1.093	0.387
	age	0.97	0.888–1.055	0.457
	African American	0.22	**0.054–0.920**	**0.038**
	Tumor grade 7+	1.98	0.395–9.913	0.406

## Discussion

We had previously demonstrated an association between obesity and advanced stage at diagnosis and biochemical failure [6]. This study examined whether there are differences in tumor microenvironment inflammation by obesity status. We did not find an association between obesity and the number of TILS and TAMS, however we did find that higher CD8 counts were associated with increased risk for biochemical failure.

Based on our findings, CD8 appears to be among the key players in determining cancer outcomes. There is, however, some discrepancy in the strength and direction of association between TILs and cancer outcomes. [23,24] Other studies have found that there are obvious differences in TIL characteristics among cancer, BPH, and healthy microenvironments [25,26]. One study showed that within the tumor microenvironment, only very low numbers of CD3+ lymphocytes were found without any evidence of cluster formation. CD8+ lymphocytes were significantly less prevalent than but showed the same distribution pattern. Healthy prostate tissue also contained sparse amounts of these TILS [23]. BPH tissue had all TILS, dispersed similarly to healthy tissue with small lymphocyte clusters observed occasionally, but fewer than in tumor tissue. There were more CD8+ cells present in BPH tissue than in healthy or carcinoma tissue [23]. Another study showed that CD8+ cells were lowest among BPH and carcinoma tissue and higher among normal and PIN tissue [24].

Most studies on PCa have investigated microenvironment infiltrates in prostatectomy or biopsy tissue. These cells typically were found to be pro-tumorigenic [27] and may be informative as part of a prognostic score [28]. Strong infiltration of CD3+ cells may be related to slow cancer progression and better prognosis of prostate cancer [23,26]. Similar to work in ovarian cancer by Preston et al. [29] and in glioblastoma by Kmiecik et al. [26], we observed a significant positive correlation between CD3 and CD8 TILS. This finding was expected, since CD8 is a sub-type of CD3 expressing TILS. TAM CD68 and CD3 also were significantly correlated in our study. Kmiecik et al. found that in multivariate analysis, CD8 was inversely associated with patient survival [26]. Our study suggested a similar relationship in that CD8 was directly associated with an adverse outcome (biochemical failure) in a multivariable model. In previous studies, higher numbers of tumor associated macrophages (TAM) were also associated with poor cancer outcomes and poor prognosis [30,31]. We observed an association between CD68 and higher tumor grade in univariate analyses, but the association with biochemical failure in multivariable analysis was not observed.

Tumor-promoting inflammation is one of the hallmarks of cancer [30,32]. Although there is controversy about the role of chronic inflammatory infiltrates in the development of potential precursors to clinically detected PCa, including proliferative inflammatory atrophy (PIA) and prostatic intraepithelial neoplasia (PIN), [33,34], the evidence is mounting that inflammation increases PCa development (via regulation of cellular events and the tumor microenvironment) and disrupts the immune response [35]. Consequently, immune cells are among the primary drivers of PCa progression. [27] The evaluation of specific inflammation cell types is important in understanding heterogeneity in the cancer microenvironment and holds promise for developing novel prognostic parameters. In particular, tumor infiltrating cells, which vary in size, distribution, and composition from patient to patient, [36] may be rendered inactive in pro-inflammatory microenvironments. [37]

### Mechanisms of Obesity

Inflammation is a key biological mechanism linking obesity to PCa progression. [9,13,15,27] Although chronic inflammation can occur in both benign and malignant prostate tissue, the state of obesity produces a systemic chronic inflammatory environment that sets the stage for cancer progression and poor prognosis. Obesity produces greater levels of systemic inflammatory factors, including adipose tissue macrophages, circulating lymphocytes, and pro-inflammatory cytokines (e.g., TNF-α, IL-6, TGF-β1, and C-reactive protein), than is produced in non-obese states [38–41]. These systemic factors trigger the release of tumor growth factors and the release of complex cellular and cytokine-related events that may stimulate inflammatory processes in the tumor microenvironment [9,18,42,43]. Obesity, therefore, holds potential to convert the tumor microenvironment to a pro-inflammatory state.

There are several other mechanisms whereby obesity can favor cancer development/progression, including hormonal changes, insulin resistance, and hypoxia-angiogenesis [42]. Related epigenetic mechanisms may limit the anti-tumor immune response by modulating both tumor and immune cells, with obesity resulting in a compromised immune system that is less capable of destroying cancer cells [23,25,44,45]. Despite this evidence for inflammation providing a link between obesity and poor PCa outcomes, it has been suggested that there may not be a relationship between the number of CD68 macrophages and BMI among breast cancer patients. [30] Similarly, we did not observe differences in the number of TILS and TAMS present in the prostate tumor microenvironment by obesity status.

Our results are critical to helping advance the scientific investigation beyond tumoral cell type and number to perhaps include cell function (there may be differences in how TILS and TAMS function by obesity status), or genetic pathways that may alter immunity/inflammation. Our results also deepen knowledge of factors that mediate obesity effects on prostate cancer. Continued research in this area will guide treatment protocols that may include immunotherapy vs. obesity reduction as they apply to diverse populations. The long-term objective is to be able to suggest ways to improve treatments for men at high risk for poor outcomes by targeting components of the tumor microenvironment. [46] Research involving obese prostate cancer patients may suggest new approaches for prostate cancer intervention via weight management, physical activity, targeted screening, and novel treatments.

### Study Limitations

#### BMI as measure of obesity

BMI measurements were readily available for this pilot study. BMI measurements are easily obtained, well-studied, and commonly cited in the literature. Studies finding associations between obesity with prostate cancer have primarily used BMI instead of other anthropometric measures. Studies also have shown that, compared to other anthropometric measures, BMI can be the strongest predictor of risk for cancer [47]. However BMI measurements may be an inadequate representation of body fat distribution. Measuring central obesity by waist circumference or visceral fat by CT scan may present a more accurate representation of obesity, better capturing and classifying patients with high amounts of adipose tissue [48].

#### Tumor assessment

Some tumor samples were insufficient and could not be evaluated. A higher percentage of these insufficient samples were from obese patients rather than from normal weight patients. More of the samples form the obese patients had been used in previous studies. We observed no differences in tumor stage/grade by obesity. However, our inability to analyze the tumors from more of the obese patients may impact the representativeness of the sample and generalizability to other phenotypically diverse populations.

#### Evaluation of cell function

Besides differences in the prevalence of TILS and TAMS, there are interesting differences in the functioning ability of these cells in tumor tissue compared to healthy tissue. These differences may be considered in future analyses. In particular, TILS appear to be mostly nonfunctional. Markers for lymphocyte effector function have been shown to be absent in tumor tissue but readily detected in healthy tissue, suggesting that the lymphocytes in tumor tissue are activated but not functionally active or perhaps suppressed in their ability to destroy tumor cells. [25] In addition, we did not consider whether nodule size is related to immune cell prevalence. However, we did examine other related tumor characteristics, such as stage and grade, in our analyses.

#### Cell types

In this pilot study, we were limited to study a relatively small number of cell types. Other factors impacting the inflammatory milieu of the tumor microenvironment include other lymphocytes and macrophages, neutrophils, mast cells, natural killer cells, and eosinophils [10]. In addition, specific cell types, such as macrophage sub-types that have been shown to vary by obesity among some cancer populations [30]. Future studies may focus on the relationship between additional cell counts, TIL ratios, and PCa outcomes by obesity status.

### Study Strengths

We used a standardized scoring system to determine cell type and count in the high tumor area of each tissue sample. Several clinical outcomes were considered in our analyses, making this research potentially translational in nature. Cancer phenotypes were combined with patient phenotypes and data about the tumor microenvironment to account for various aspects of cancer biology that may be most relevant to treatment outcomes. [32]

Research into the effects of obesity on lymphocytes has produced conflicting results due to cross sectional studies and small sample size. However, many of these studies have focused on circulating lymphocyte levels. [41] Our study, although relatively small and with some short follow-up times, had a prospective component in that we were able to connect microenvironment inflammation at the time of the prostatectomy to later biochemical failure. We also accounted for some co-morbidities that may influence inflammatory processes and prostate cancer outcomes. Much of the work related to obesity, immune cells, and cancer has focused on other cancer populations, such as women with breast cancer. To date, our study is the first to examine these relationships among a cohort of obese and non-obese PCa patients. Because cancer occurs in *in vivo* environments, we studied these relationships in prostatectomy specimens in order to provide a clearer picture of the dynamics that may reflect the human tumor environment. [32] In future studies, these microenvironment factors may also be studied with genetic signatures of the tumor in order to get a better understanding of gene-microenvironment interactions and to identify patients at highest risk for poor outcomes.

## Conclusions

It is important to study factors that modify inflammation processes and patterns so that novel treatment targets and modalities can be developed to inhibit the growth and metastasis of cancer cells. [13,15] Because there is heterogeneity within the malignant tumor, describing the inflammatory response during carcinogenesis may provide a more complete understanding of the molecular pathogenesis of the cancer. [49] The quantification of changes in the tumor microenvironment may provide tumor signatures, or “microenvironment signatures” [50], to improve cancer prognostics that can be applied to other cancers, particularly to identify high risk patients. While immune cell types are divided into functional subsets, their prevalence and functional status is often shaped by the tumor microenvironment. [27] Additional research is needed to identify specific mediators and moderators of poor PCa outcomes using relevant models of progression, strategies for inflammation prevention, and interventions with targeted therapies.

Our results indicate that tumor infiltrating lymphocytes and macrophages do not differ by obesity status. However, “healthy” obese individuals might impose a bias in studies aimed at looking at the impact of the pro-inflammatory state on tumors. Nonetheless, **t**his pilot study laid a foundation for extending this research to include various BMI groups and other ethnic populations for which inflammatory responses may be more pronounced. By studying patients reflecting a cross-section of BMIs, we were also able to distinguish factors that increase risk from those that are protective in individuals with different phenotypes. Research involving patient BMI may suggest new approaches for chronic disease intervention via weight management, physical activity, targeted screening of high risk groups and novel treatments using agents that target inflammation pathways. If patients can be better classified in terms of risk for disease progression, treatments can be tailored to prevent over- or under-treatment of PCa patients. [51] In this era of personalized medicine, a thorough understanding of biological mechanisms as well as potential modifiers of cancer progression (including assessment of lifestyle factors) [32] is needed to develop therapeutic and public health strategies that are suitable for individuals of various genotypes and phenotypes.

## Supporting Information

S1 File SCORE Final Dataset. (XLS)
